# Efficacy and safety of branded vs generic lacosamide in epilepsy: a retrospective real-world study

**DOI:** 10.1007/s10072-025-08563-3

**Published:** 2025-10-11

**Authors:** Giuseppe Salafica, Diana Tilenni, Attilio Vinaccia, Giovanni Tripepi, Chiara Martellino, Salvatore Maria Lima, Giorgia Atanasio, Fabio Lamanna, Orazio Pardeo, Mariangela Panebianco, Angelina Laganà, Angelo Labate

**Affiliations:** 1 Neurophysiopatology and Movement Disorders Clinic, Department of Clinical and Experimental Medicine, University of Messina, Messina, Italy; 2 Neurology Clinic, University of Palermo, Palermo, Italy; 3 National Research Council (CNR), Institute of Clinical Physiology (IFC), Clinical Epidemiology of Renal Diseases and Hypertension, Ospedali Riuniti, Reggio Calabria, Italy; 4 Neurology and Stroke Unit-ARNAS Garibaldi, Catania, Italy; 5 Department of Biomedicine, Neuroscience, and Advanced Diagnostic (BIND), University of Palermo, Palermo, Italy

**Keywords:** Epilepsy, Lacosamide, Generic formulation, Real-world study

## Abstract

**Purpose:**

Lacosamide (LCS) is a third-generation antiseizure medication (ASM) approved for focal-onset seizures and generalized epilepsy. Although the branded formulation, Vimpat^®^, has shown efficacy and safety, the introduction of generic versions, such as Stutan^®^, raises concerns about clinical equivalence, especially considering the potential for therapeutic fluctuations that could result in breakthrough seizures or adverse events. This study aimed to compare the real-world efficacy, safety and tolerability of branded lacosamide (Vimpat^®^) versus its generic counterpart (Stutan^®^) in patients with focal or generalized epilepsy.

**Methods:**

A multicenter, retrospective, observational study was conducted at two epilepsy centers in Southern Italy. Sixty adult patients were included and divided into two groups: Group A (*n* = 30) received branded LCS and Group B (*n* = 30) received the generic formulation. Data were collected at treatment initiation (T0) and the first follow-up (T1), including seizure frequency, adverse events and dose adjustments. The primary outcome was the responder rate (≥ 50% reduction in seizure frequency), with secondary outcomes including seizure freedom, adverse events and dose changes.

**Results:**

Baseline characteristics were similar between groups. The average daily LCS dose was significantly higher in the Vimpat^®^ group (275 ± 121 mg) compared to the Stutan^®^ group (168 ± 89 mg, *p* < 0.001). Despite this, efficacy outcomes were comparable, with 60.0% of patients in Group A and 43.3% in Group B achieving a ≥ 50% seizure reduction (*p* = 0.08). Adverse events were mild or moderate.

**Conclusions:**

In this real-world setting, generic LCS (Stutan^®^) demonstrated comparable efficacy, safety and tolerability to Vimpat^®^, supporting its clinical use as a valid alternative in epilepsy management.

## Introduction

Epilepsy is a chronic neurological disorder characterized by recurrent unprovoked seizures, affecting approximately 50 million people worldwide, with significant impact on physical, psychological and social well-being [1, 2]. Despite the availability of numerous antiseizure medications (ASMs), around one-third of patients continue to experience uncontrolled seizures, underlining the need for optimized pharmacological strategies [3].

A generic drug is a version of a brand-name drug that contains the same active ingredient, but is typically sold at a lower price once the patent expires. The use of generic drugs is steadily increasing and recent studies show that generic ASMs offer high safety when switching between different products, minimizing the risk of clinically significant variations in plasma concentrations [4].

Lacosamide (LCS) is a third-generation ASM approved for the treatment of focal-onset seizures in both monotherapy and add-on settings. It has also shown efficacy in some forms of generalized epilepsy [5, 6]. LCS acts by selectively enhancing the slow inactivation of voltage-gated sodium channels, thereby stabilizing neuronal membranes without affecting physiological fast inactivation [7]. This mechanism may contribute to its favorable tolerability and lower risk of cognitive side effects compared to other ASMs [8, 9]. The branded version of LCS (Vimpat^®^) has been extensively evaluated in randomized controlled trials and real-world clinical studies, demonstrating consistent efficacy and safety in various patient populations [10, 11]. As with many ASMs, the expiration of the original patent has led to the introduction of generic formulations such as Stutan^®^, raising questions regarding therapeutic equivalence in clinical practice. Generic drugs are approved based on bioequivalence, which means they must have similar absorption levels (like Cmax and AUC) to the original drug, usually within a ± 20% range. However, these laboratory measures do not always guarantee the same clinical results, especially for drugs like antiseizure medications (ASMs), which have a narrow margin between effective and harmful doses [4, 12].

Concerns about switching from branded to generic ASMs are not new. In epilepsy, even small variations in drug exposure may result in breakthrough seizures or adverse effects, potentially compromising seizure control and patient safety [13]. Some clinicians and patients express reluctance to switch; several reports and studies have highlighted cases of seizure recurrence, worsening control, or side effects following such switches, raising concerns about the reliability and safety of generic substitution in epilepsy treatment [14, 15]. On the other hand, multiple studies suggest that generic ASMs are generally safe and effective when bioequivalence is confirmed and offer important advantages in terms of cost and accessibility [16].

Therefore, the real-world comparative studies are essential to evaluate whether generic drugs provide the same effectiveness and safety like the brand-name version. Although some recent studies suggest that branded and generic LCS can be used in the same way [17], more real-world data are needed to help medical doctors to do it without worries.

In this context, we aimed to compare the efficacy, safety and tolerability of branded LCS (Vimpat^®^) and its generic equivalent (Stutan^®^) in patients with focal or generalized epilepsy across two epilepsy centers in Southern Italy.

## Materials and methods

We conducted a multicenter, retrospective, observational study at two epilepsy centers in Sicily, within the context of routine clinical practice. The aim of the study was to compare the efficacy and tolerability of branded LCS (Vimpat) versus its generic equivalent (Stutan) in patients with focal or generalized epilepsy. Patients treated with Vimpat were recruited between 2007 and 2025, while those treated with Stutan were recruited from 2021 to 2025. A total of 60 patients were included and divided into two treatment groups:

Group A (*n* = 30): Patients treated with branded LCS (Vimpat);

Group B (*n* = 30): Patients treated with generic LCS (Stutan).

Patients included in the study were diagnosed with focal epilepsy, generalized epilepsy or epilepsy of unknown type, according to the 2017 classification of the International League Against Epilepsy (ILAE) [18]. Patients were treated with LCS either as monotherapy or as part of combination therapy with other antiseizure medications (ASMs) and had to be on treatment for at least 6 months.

Exclusion criteria included: pregnancy or breastfeeding, known allergy to LCS, and non-adherence to treatment. A complete clinical and instrumental workup was performed for all patients, including electroencephalogram (EEG), brain magnetic resonance imaging (MRI) and detailed neuropsychological assessment. Collected variables included: age, sex, age at epilepsy onset, epilepsy duration (in years), seizure type (focal, generalized, focal to bilateral, unknown), epilepsy type (focal, generalized, unknown), epilepsy etiology (structural, genetic, metabolic, autoimmune, infectious, unknown), intellectual disability (present/absent), psychiatric comorbidities (present/absent), presence of vagus nerve stimulation (VNS), history of epilepsy surgery, interictal EEG findings (normal, focal abnormalities, generalized abnormalities), LCS daily dose (mg/day), monthly seizure frequency and concomitant ASMs, as detailed in Table 1.

**Table 1:** Demographic data and clinical characteristics of all patients at T0

	GROUPS		
T0	VIMPAT (*n* = 30)	STUTAN (*n* = 30)	**P value**
Age	46(± 18)	53(± 15)	0.11
Sex (f%) (m%)	13(43.3) 17(56.7)	19(63.3) 11(36.7)	0.20
Age at epilepsy onset	33(± 19.4)	34(± 20.0)	0.92
Epilepsy duration (years)	11(4.8–20)	13(5–30)	0.38
Seizure type (%) 1 = focal onset 2 = generalized 3 = unknown 4 = focal to bilateral	7(23.3) 4(13.3) 3(10.0) 16(53.3)	12(40.0) 2(6.7) 3(10.0) 13(43.3)	0.51
Epilepsy type (%) 1 = focal epilepsy 2 = generalized epilepsy 3 = unknown	20(66.7) 6(20.0) 4(13.3)	20(66.7) 4(13.3) 6(20.0)	0.70
Epilepsy etiology (%) 1 = structural 2 = genetic 3 = metabolic 4 = autoimmune 5 = infectious 6 = unknown	14(46.7) 1(3.3) 15(50.0)	10(33.3) 20(66.7)	0.30
Intellectual disability (%) absent present	23(76.7) 7(23.3)	28(93.3) 2(6.7)	0.07
Psychiatric comorbidities (%) Absent Present	23(76.7) 7(23.3)	27(90.0) 3(10.0)	0.17
VNS (%) No Yes	29(96.7) 1(3.3)	30(100)	0.31
Epilepsy surgery (%) No Yes	30(100)	30(100)	…
Interictal EEG findings (%) 0 = normal 1 = focal abnormalities 2 = generalized abnormalities	8(26.7) 12(40.0) 10(33.3)	10(33.3) 13(43.3) 7(23.4)	0.67
Dosage (mg/die)	275(± 121)	168(± 89)	< 0.001
Monthly seizure frequency before LCS	3.1(± 6.4)	2(± 3)	0.88
ASMs concomitant(%) 0 1 2 3	7(23.3) 11(36.7) 11(36.7) 1(3.3)	10(33.3) 11(36.7) 9(30.0)	0.63

Abbreviations: *LCS* Lacosamide, *ASMs* Anti-Seizure Medications, *T0* Time 0

Retrospective data were collected on seizure frequency and the occurrence of adverse events. The time of lacosamide introduction was defined as T0, while T1 corresponded to the first follow-up evaluation. Seizure frequency was primarily derived from patient diaries and confirmed through clinical charts and regular follow-up visits. Efficacy outcomes included the responder rate (≥ 50% reduction in seizure frequency) and sustained seizure freedom, defined as the absence of epileptic episodes for at least 12 consecutive months. A ≥ 50% reduction was calculated by comparing the mean seizure frequency in the 12 months following treatment initiation with that of the 12 months prior.

Safety outcomes referred to the frequency, type and severity of adverse events (AEs). Table 2.

**Table 2:** Efficacy and Safety outcomes of all patients at T1

	GROUPS		
T1	VIMPAT (*n* = 30)	STUTAN (*n* = 30)	**P**
Time between LCS initiation and follow-up (months)	12(6–90)	12(6–39)	0.29
Seizures/month	2.7(± 6.4)	1.6(± 2.8)	0.47
	7(23.3) 18(60.0) 5(16.7)	4(13.4) 13(43.3) 13(43.3)	0.08
Action on medication (%) 0 = Maintain therapy 1 = Suspend or reduce 2 = Increase dosage	24(80.0) 4(13.3) 2(6.7)	23(76.7) 2(6.7) 5(16.6)	0.37
Drug dosage	282(± 105)	183(± 85)	< 0.001
ASMs concomitant (%) 0 1 2 3	6(20.0) 13(43.3) 11(36.7)	13(43.3) 12(40.0) 4(13.4) 1(3.3)	0.08
Adverse events (%) No Si	28(93.3) 2(6.7)	29(96.7) 1(3.3)	1.00
Adverse event severity (%) 0 = Mild 1 = Moderate 2 = Severe	1(50.0) 1(50.0)	1(100)	1.00
Outcome adverse events (%) 1 = resolved 2 = resolved with sequelae 3 = persists	2(100)	1(100)	…
Action taken (%) 0 = none 1 = reduction in LCS dosage 2 = suspension of LCS	1(50) 1(50)	1(100)	0.22

Abbreviations *LCS* Lacosamide, *ASMs* Anti-Seizure Medications, *T*1 Time 1

### Statistical analysis

Descriptive statistics were used to summarize baseline demographic and clinical characteristics. Continuous variables were reported as means with standard deviations (SD) or medians with ranges, depending on data distribution, and compared between groups using the Student’s t-test or the Mann–Whitney U test, as appropriate. Categorical variables were expressed as frequencies and percentages, and group comparisons were performed using the chi-square test or Fisher’s exact test when expected cell counts were below five. A two-tailed p-value of less than 0.05 was considered statistically significant. Statistical analyses were conducted using STATA 16 (StataCorp. 2025. Stata Statistical Software: Release 19. College Station, TX: StataCorp LLC).

## Results

A total of 60 patients were enrolled, who began treatment with either Vimpat or Stutan between 2007 and 2025. Of the 60 patients, 30 were assigned to receive Vimpat (Group A), while 30 received Stutan (Group B). The demographic and clinical characteristics at baseline (T0) are summarized in Table 1.

The mean age of patients was 46 (± 18) years for Group A and 53 (± 15) years for Group B (*p* = 0.11). The gender distribution was as follows: 43.3% females and 56.7% males in Group A, and 63.3% females and 36.7% males in Group B (*p* = 0.20). The mean age at epilepsy onset was 33 (± 19.4) years for Group A and 34 (± 20.0) years for Group B (*p* = 0.92). The average duration of epilepsy was 11 (4.8–20) years for Group A and 13 (5–30) years for Group B (*p* = 0.38).

According to the ILAE 2017 classification epilepsy was distinguished in: structural, genetic, infective, metabolic, immune-related and unknown causes. In Group A, 14 (46.7%) patients had a diagnosis of structural epilepsy, compared to 10 (33.3%) in Group B (*p* = 0.30). Only one patient in Group A (3.3%) had a genetic etiology. The number of cases with unknown origin was 15 (50.0%) in Group A and 20 (66.7%) in Group B (*p* = 0.30).

At baseline, the distribution of seizure types was as follows: in Group A, seven patients (23.3%) had focal seizures, four (13.3%) had generalized seizures, three (10%) had unknown seizures and 16 (53.3%) had focal seizures evolving to bilateral seizures. In Group B, 12 patients (40.0%) had focal seizures, two (6.7%) had generalized seizures, three (10%) had unknown seizures and 13 (43.3%) had focal to bilateral seizures (*p* = 0.51).

Intellectual disability was observe only in 7 patients (23.3%) in Group A and in 2 patients (6.7%) in Group B (*p* = 0.07).

Psychiatric comorbidities were present in 23.3% of patients in Group A and 10% in Group B (*p* = 0.17). Only one patient from Group A underwent VNS implantation (*p* = 0.31). No patients had undergone epilepsy surgery.

Interictal EEG results showed that eight patients (26.7%) in Group A and 10 patients (33.3%) in Group B had normal EEG findings. In contrast, 12 patients (40%) in Group A and 13 patients (43.3%) in Group B exhibited focal abnormalities, while 10 patients (33.3%) in Group A and 7 patients (23.4%) in Group B had generalized abnormalities (*p* = 0.67). The mean daily dose of LCS was 275 (± 121) mg for Group A (Vimpat) and 168 (± 89) mg for Group B (Stutan) (*p* < 0.001).

Before starting LCS therapy, Group A reported a mean of 3.1 (± 6.4) seizures for month, while Group B reported a mean of 2 (± 3) seizures for month (*p* = 0.88).

The distribution of concomitant ASMs was as follows: in Group A, 7 patients (23.3%) did not take any concomitant ASMs, 11 patients (36.7%) took one, 11 patients (36.7%) took two and 1 patient (3.3%) took three ASMs. In Group B, 10 patients (33.3%) did not take any concomitant ASMs, 11 patients (36.7%) took one, 9 patients (30%) took two and no patients took three ASMs (*p* = 0.63).

The average time between the initiation of lacosamide therapy and the follow-up was 12 months (6–90) for Group A and 12 months (6–39) for Group B (*p* = 0.29).

In Group A, 1 patient reported a mild adverse event and 1 patient reported a moderate adverse event. In response, no action was taken for the mild adverse event, while the LCS dose was discontinued for the patient with the moderate adverse event. In Group B, 1 patient reported a mild adverse event and the dose of LCS was reduced. All patients experienced full resolution of their adverse events.

**Fig. 1:**
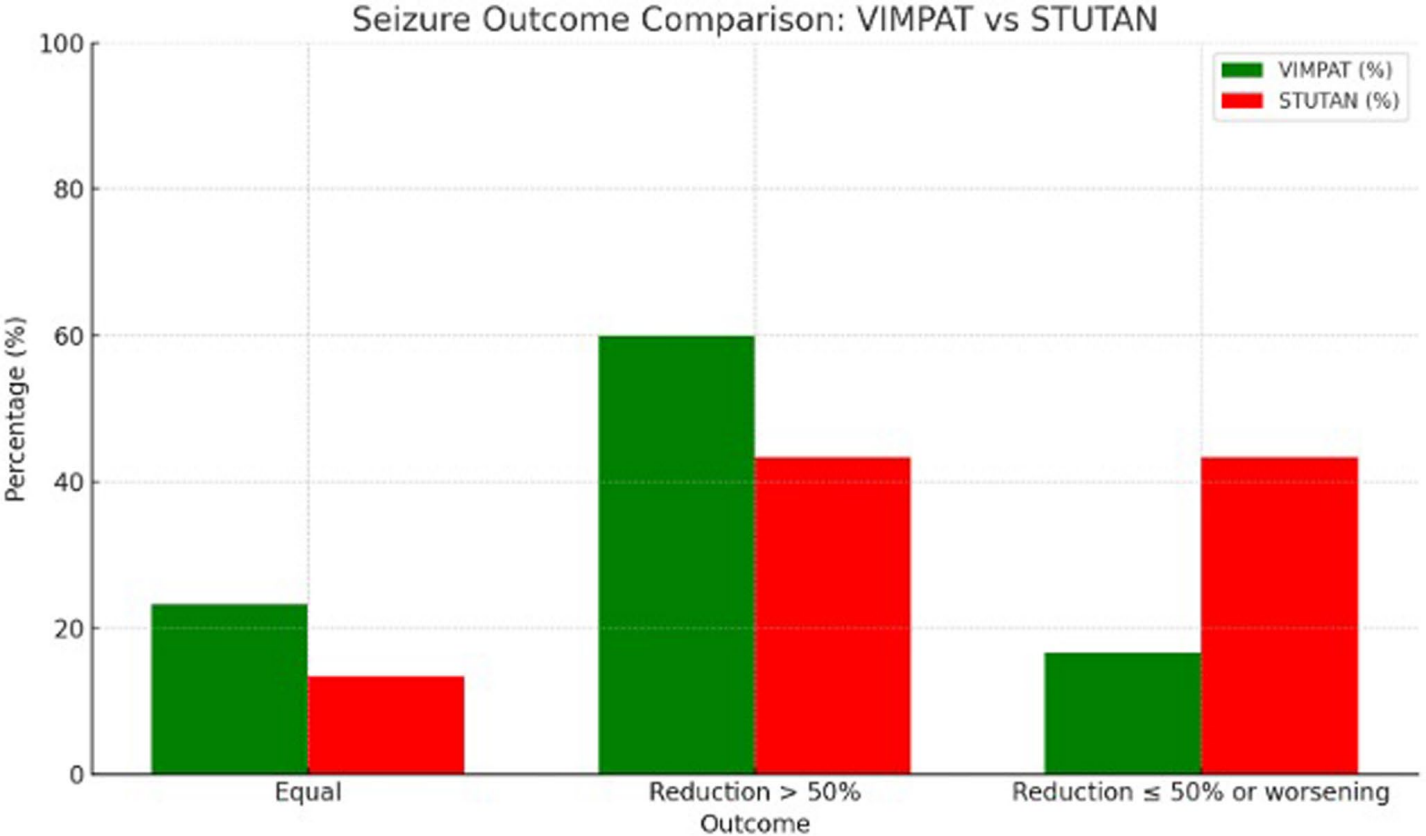
Seizure outcome comparison: Vimpat vs Stutan

The primary study outcomes are shown in the Fig. 1 as follows: in Group A, 7 patients (23.3%) showed no change in seizure frequency, 18 patients (60.0%) demonstrated a reduction of over 50% and 5 patients (16.7%) had a reduction of less than 50% or experienced worsening. In Group B, 4 patients (13.4%) showed no change, 13 patients (43.3%) had a reduction greater than 50% and 13 patients (43.3%) had a reduction of less than 50% or worsening of seizures (*p* = 0.08).

At the first follow-up, in Group A, 24 patients (80.0%) continued with unchanged therapy, 4 patients (13.3%) had a dose reduction or discontinuation and 2 patients (6.7%) had a dose increase. In Group B, 23 patients (76.7%) maintained unchanged therapy, 2 patients (6.7%) had a dose reduction or discontinuation and 5 patients (16.6%) had a dose increase (*p* = 0.37). Table 1.

## Discussion

In the current study our findings suggest that LCS is a very good and safe therapeutic option in patients with epilepsy regardless the use of branded or generic type of LCS [19–21].

The results of this retrospective study, conducted at two epilepsy centers in Sicily (University Hospital of Messina and ARNAS Garibaldi of Catania), involving 60 patients treated with LCS (30 in Group A, who received Vimpat, and 30 in Group B, who received Stutan), suggest that Stutan is comparable to Vimpat in terms of efficacy, tolerability and safety in the management of epilepsy [17].

A fundamental aspect of this study is the comparability between the two treatment groups. Statistical analyses showed that the mean age, sex distribution, age at epilepsy onset and disease duration were similar, with no statistically significant differences. This confirms the good homogeneity between the groups and allows for the exclusion of biases related to the baseline demographic and clinical characteristics of the patients. In this context, we can affirm that the groups were well balanced and the differences in observed outcomes between the groups are mainly attributable to the treatments administered. Furthermore, the etiology of epilepsy in both groups was similarly distributed, with a predominance of cases of unknown origin (50% and 66.7% in Groups 1 and 2, respectively), ensuring good comparability between the groups in terms of epilepsy causes. Focal seizures were the most common seizure type in both groups, with a higher frequency in Group B, but without statistically significant differences between the groups.

An important result concerns the frequency of seizures before and after treatment. Although patients in Group A reported an average of 3.1 seizures for month compared to 2.0 in Group B at baseline, the mean reduction in seizures during follow up was similar between the two groups. In Group A, 60% of patients showed a reduction in seizures greater than 50%, compared to 43.3% in Group B [19]. These data suggest that, despite a slight difference in seizure reduction between the two groups, Stutan’s efficacy is comparable to that of Vimpat.

The difference in the mean daily dose of lacosamide (275 mg for Vimpat vs. 168 mg for Stutan) was significant, but it did not negatively impact the efficacy outcomes between the groups. This suggests that, despite the use of higher doses of Vimpat, the efficacy of Stutan is comparable, likely due to its bioequivalence [17]. This further supports the idea that Stutan represents an equally valid therapeutic option compared to Vimpat, particularly considering the lower cost of the generic drug [22].

The tolerability of the treatment was analyzed by monitoring adverse events. Only a small percentage of patients reported adverse events and their intensity was generally mild or moderate. Most adverse events resolved without sequelae and no significant differences were observed between the two groups regarding the intensity and outcome of adverse events. Additionally, the follow-up showed that most patients maintained the same dose of lacosamide, indicating good overall tolerability [23].

In terms of therapeutic adjustments, patients in Group A showed a greater tendency to maintain the same dosage compared to patients in Group B. This could be due to a difference in the initial dosage or response to treatment, with patients in Group B possibly requiring more frequent therapeutic adjustments. Overall, the results suggest that Stutan presents similar efficacy and a comparable safety and tolerability profile to Vimpat [17].

The study has some limitations. As a retrospective analysis, there is a potential for bias in patient selection and data recording, which could have affected the perceived effectiveness of the treatment. However, this limitation is mitigated by the inclusion of multiple centers and, being a real-world study, it reflects the actual clinical practice, where patient selection and data collection are influenced by the diversity and complexity of everyday healthcare settings, adding to the validity of the findings. Additionally, the follow-up duration exhibited some variability within each group. This variation may have influenced the consistency of the results, but it also reflects the inherent flexibility of real-world clinical practice, where follow-up schedules can vary based on individual patient needs and healthcare provider preferences. The disparity in the gender distribution between the two groups (43% vs. 63% women) is a limitation of our research. Given the relatively small sample size, we were not able to reliably assess the potential influence of gender on treatment response or tolerability. Another limitation is the notable difference in dosages between the groups. While this difference in dosage could potentially influence treatment outcomes and it might have influenced clinical outcomes, it is important to consider that the efficacy results were comparable between the groups despite the dosage discrepancy. This suggests that the therapeutic effect of Stutan^®^ is not heavily reliant on higher dosages, supporting the clinical equivalence of the two formulations. In real-world clinical practice, dosage adjustments are often made based on individual patient needs and tolerability, making the observed difference in dosage less of a concern in evaluating the overall effectiveness of the treatments. Future prospective studies with larger sample sizes and dose-adjusted analyses will be needed to achieve sufficient statistical power and to more definitively clarify these issues.

## Conclusions

This study highlights the potential of generic LCS (Stutan^®^) as a successful viable treatment option in clinical practice. The consistent outcomes observed across diverse patient profiles support its broader use in epilepsy management, particularly in healthcare settings where economic sustainability is a consideration [4].

Nonetheless, further prospective and randomized studies are needed to confirm these results and assess potential variables not captured in this retrospective study.

## Data Availability

All data generated or analyzed during this study are included in this published article.
